# A Low-Phenylalanine-Containing Whey Protein Hydrolysate Stimulates Osteogenic Activity through the Activation of p38/Runx2 Signaling in Osteoblast Cells

**DOI:** 10.3390/nu14153135

**Published:** 2022-07-29

**Authors:** Tingting Bu, Yuting Ren, Songfeng Yu, Jiexia Zheng, Ling Liu, Peilong Sun, Jianping Wu, Kai Yang

**Affiliations:** 1 Department of Food Science and Technology, Zhejiang University of Technology, Hangzhou 310014, China; butingting@zjut.edu.cn (T.B.); renyuting74@163.com (Y.R.); sun_pl@zjut.edu.cn (P.S.); 2 College of Biosystems Engineering and Food Science, Zhejiang University, Hangzhou 310027, China; sfyu9002@zju.edu.cn (S.Y.); jxzheng@zju.edu.cn (J.Z.); lingliu21@zju.edu.cn (L.L.); 3 Department of Agricultural, Food and Nutritional Science, University of Alberta, Edmonton, AB T6G 2R3, Canada; jwu3@ualberta.ca

**Keywords:** phenylketonuria, whey protein hydrolysate, osteoblasts, differentiation, runt-related transcription factor 2, p38 mitogen-activated protein kinase

## Abstract

A phenylalanine (Phe)-restricted diet is indispensable for individuals suffering from phenylketonuria (PKU). Our previous study reported a low-Phe-containing whey protein hydrolysate (LPH) prepared from a selected whey protein hydrolysate (TA2H). This study aimed to investigate the osteogenic activity of LPH and TA2H in MC3T3-E1 preosteoblast cells and explore the underlying mechanism. Results showed that the treatment of TA2H and LPH (at the final concentrations of 100–1000 μg/mL) had a stimulatory effect on the proliferation, differentiation, and mineralization of MC3T3-E1 cells. The LPH of 1000 μg/mL significantly increased cell proliferation (2.15- ± 0.11-fold) and alkaline phosphatase activity (1.22- ± 0.07-fold), promoted the protein and mRNA levels of runt-related transcription factor 2 (Runx2, 2.50- ± 0.14-fold and 2.97- ± 0.23-fold, respectively), enhanced the expression of differentiation biomarkers (type-I collagen, osteocalcin, and osteopontin), increased calcium deposition (1.56- ± 0.08-fold), and upregulated the ratio of osteoprotegerin/receptor activator of nuclear factor-κB ligand. The exploration of signaling pathways indicated that the activated p38-dependent Runx2 signaling contributed to the LPH-induced osteogenesis. These results provided evidence, for the first time, that a prepared low-Phe whey protein hydrolysate positively modulated the activity of osteoblasts through the p38/Runx2 pathway, thereby providing a new osteoinductive protein substitute to make functional PKU food.

## 1. Introduction

Phenylketonuria (PKU) is a recessively inherited disorder caused by the mutation of the gene coding for phenylalanine hydroxylase (PAH, EC 1.14.16.1), and it results in the inability to catalyze phenylalanine (Phe) into tyrosine [1]. If an untreated typical PKU patient (BH_4_ deficiency) fails to control Phe, the excessive Phe and its metabolites would accumulate in blood and tissues, causing irreversible neurological damage and mental retardation [2]. The dietary nitrogen requirements of PKU individuals should be met through low-protein diets and Phe-free medical foods [3]. However, long-standing dietary adherence to a Phe-restricted diet might increase the risk of suboptimal nutritional status for PKU individuals [4].

Reduced bone mineral density (BMD) has been reported as a chronic complication in PKU individuals [5]. Individual studies indicated that PKU patients exerted lower BMD and higher risk of fracture [6,7,8]; however, systematic reviews and meta-analyses summarized that the reduced BMD in PKU individuals was not clinically evident according to the standard definitions of low BMD [9,10,11]. Imbalanced bone remodeling, specifically activated spontaneous osteoclastogenesis and bone resorption, has been observed in the isolated blood cells of PKU individuals [12,13].

The skeleton is a highly dynamic organ under constant metabolism (or remodeling). The delicate balance between bone formation and bone resorption is regulated by the synergistic actions of osteoblasts and osteoclasts [14]. Bone formation is the final and longest stage of bone remodeling that involves new bone formation and mineralization. Osteoblasts not only act as critical anabolic bone cells but also mediate the dynamic process of bone resorption by secreting the receptor activator of nuclear factor-κB ligand (RANKL) and its soluble decoy receptor osteoprotegerin (OPG) [15]. Therefore, developing new protein-sourced substitutes which could promote osteogenic activity is beneficial for the nutritional and physiological management of PKU patients, especially for children and adolescents.

Whey protein, a portion of milk protein with highly nutritional and physiological functions, has been widely used as food ingredient. Our previous study summarized that whey protein as a whole or its individual active components, as well as whey hydrolysates and whey-derived bioactive peptides, have positive roles in bone health, especially bone formation [16]. Whey protein hydrolyzed by alcalase/protamax/flavourzyme stimulated osteogenic activity in osteoblast cells and growing rats [17]. Lactoferrin (LF), a multifunctional protein occurring in whey, was identified as an effector molecule in skeletogenesis [18]. Three whey-derived peptides (YVEEL, YLLF, and WLAHK) induced the expression of osteoblast differentiation marker genes; in addition, two whey-derived peptides (YVEEL and YLLF) attenuated bone loss in ovariectomized rats by suppressing inflammatory cytokines and enhancing bone formation markers [19,20]. Hence, whey is a promising source of osteogenic peptides.

Mitogen-activated protein kinase (MAPK) cascades and phosphatidylinositol 3-kinase/protein kinase B (PI3K/Akt) signaling are involved in skeletal development and bone homeostasis [21,22]. Runt-related transcription factor 2 (Runx2) acts as a master transcription factor in regulating bone formation [23]. The interaction among Runx2, MAPK, and PI3K/Akt signaling mediates osteogenesis. To date, several food-derived peptides have been reported to modulate osteoblast differentiation via the Akt/Runx2 pathway [24] and p38/Runx2 pathway [25].

In our previous work, a low Phe-containing hydrolysate (LPH) was prepared from a selected whey protein hydrolysate (TA2H) using macroporous resin adsorption, which could be used as a protein substitute to make PKU foods [26]. Moreover, the LPH showed anti-osteoporotic potential in an ovariectomized mice model through stimulating osteogenesis and inhibiting osteoclastogenesis [27]; however, the inner mechanism of LPH is still unclear. Therefore, the current study aimed to investigate the effects of the selected whey protein hydrolysate and its derived LPH on the proliferation, differentiation, and mineralization in MC3T3-E1 osteoblast cells. This work also aimed to further explore the involvement of MAPK and Akt signaling pathways in LPH-stimulated osteoblast differentiation.

## 2. Materials and Methods

### 2.1. Materials and Chemicals

Whey protein concentrate 80% (WPC-80) powder was donated by Beingmate Food Co., Ltd. (Hangzhou, China). D101 macroporous resin was purchased from Macklin Biochemical Technology Co., Ltd. (Shanghai, China). Thermoase C100 (900,000 U/g) and Protease A2SD (100,000 U/g) were kindly provided by Amano Enzyme Inc. (Nagoya, Japan). Alpha minimum essential medium (α-MEM), fetal bovine serum (FBS), penicillin–streptomycin, and trypsin-EDTA (0.25%) were purchased from Hyclone/Thermo Fisher (Waltham, MA, USA). Ascorbic acid and β-glycerophosphate of cell culture grade, and dimethyl sulfoxide (DMSO) were bought from Sigma-Aldrich Co., Ltd. (St. Louis, MO, USA). All other chemicals were of analytical grade and were purchased from Sinopharm Chemical Reagent Co. (Shanghai, China).

### 2.2. Preparation of Whey Protein Hydrolysate (TA2H) and Its Derived Low-Phe Hydrolysate (LPH)

The preparation of TA2H and LPH was carried out according to our previous method [23]. Briefly, WPC-80 powder was dissolved in deionized water at 5 g/100 mL and was sequentially hydrolyzed by an endopeptidase Thermoase C100 (pH 7.5, 65 °C) and an exopeptidase Protease A2SD (pH 7.0, 50 °C) for 3 h at a ratio of 1% (*w/w*, enzyme/substrate), respectively. The supernatant whey protein hydrolysate (defined as TA2H) was further subjected to a D101 macroporous resin column to remove Phe. The fraction of eluant was collected, concentrated, lyophilized, and defined as low-Phe hydrolysate (LPH). The amino-acid compositions were measured using an amino-acid analyzer (LC-16AAA, SHIMADZU, Kyoto, Japan). TA2H and LPH samples used for cell experiments were desalted with 50% (*v/v*) acetonitrile/deionized water using Sep-Pak C18 cartridges (30 cc, 10 g sorbent, Waters, Milford, MA, USA) and were further sterilized through 0.22 μm filters (PES, Merck Millipore, Billerica, MA, USA). The peptide concentrations were determined using a bicinchoninic acid (BCA) protein assay kit (Beyotime, Nanning, China).

### 2.3. Cell Culture

Preosteoblast cell line MC3T3-E1 (subclone 4, CRL-2593) was purchased from ATCC (Manassas, VA, USA) and was incubated in growth medium (α-MEM medium containing 10% FBS and 1% penicillin/streptomycin) at 37 °C under 5% CO_2_ atmosphere. After reaching 80–90% confluency, E1 cells were subcultured into culture plates using trypsin-EDTA (0.25%). The cells were only cultured in differentiation medium (growth medium supplemented with 50 μg/mL ascorbic acid and 10 mM β-glycerophosphate) for the mineralization assay.

### 2.4. Cell Proliferation Assay

The cell proliferation assay was as described by Cornish et al. [28]. MC3T3-E1 preosteoblasts were seeded in 96-well plates at a density of 2 × 10^3^ cells per well in growth medium. After 24 h of growth to semiconfluency, the medium was changed with serum starvation medium (α-MEM containing 0.1% BSA) for 12 h. Then, the cells were treated differently: (1) control group, serum starvation medium; (2) sample groups, different concentrations of TA2H and LPH (10, 100, 500, and 1000 μg/mL) prepared in serum starvation medium. After 24 h or 48 h of growth, 10 μL of CCK-8 reagent was added to each well, before incubation at 37 °C for 1 h and absorbance detection by a microplate reader (Multiskan FC, Thermo Fisher, Waltham, MA, USA). Cell proliferation was represented as absorbance at 450 nm.

### 2.5. ALP Activity Assay

The cells were seeded on six-well plates at a density of 1 × 10^5^ cells per well and incubated in growth medium. After the cells reached ~85% confluency, cells were treated with growth medium only (control group) or growth medium containing TA2H and LPH (10, 100, 500, and 1000 μg/mL, sample groups) for 72 h to induce differentiation, according to the method of Shang and Wu [29]. Then, cells were washed with PBS three times and lysed in RIPA lysis buffer (without inhibitors, Beyotime) to prepare cell lysates. The protein content was measured using a BCA protein assay kit. The ALP activity was evaluated using an ALP assay kit (Beyotime) and was represented as the unit activity per mg of protein content in the cell lysate. One unit of the ALP activity corresponded to the production of 1 μmol of *p*-nitrophenol per min at 37 °C.

### 2.6. ELISA Assay

The cells were cultured as described in the ALP assay and were treated with growth medium only (control group) or growth medium containing TA2H and LPH (10, 100, 500, and 1000 μg/mL, sample groups) for 3 or 6 days. The medium was changed every 3 days. After incubation, the collected culture medium after 3 days was used for the detection of type-I collagen (COLI) and RANKL, while the collected culture medium after 6 days was measured for osteocalcin (OCN) and osteopontin (OPN). The indices were measured using mouse ELISA kits (Meibiao Biological Co., Nanjing, China) according to the manufacturer’s instructions and were calculated according to standard curves.

### 2.7. Real-Time Polymerase Chain Reaction (RT-PCR) Assay

The cells were cultured as described in ELISA assay. Total RNA was extracted using Trizol Reagent (Beyotime) and was further reverse-transcribed into cDNA using a PrimeScript RT reagent Kit with gDNA Eraser (TaKara, Beijing, China). Gene-specific primer sequences of Runx2, COLI, OCN, OPN, and β-actin are shown in Table 1. Each PCR reaction was carried out in a 96-well PCR microplate using a CFX96TM real-time PCR instrument (Bio-rad, Hercules, CA, USA). The expression levels of target genes were calculated and normalized to the reference gene β-actin.

### 2.8. Western Blot Analysis

The cells were cultured as described in the ALP assay. The harvested cell lysates after 3 days were measured using a BCA protein assay. Equal amounts of proteins (15–30 μg) were separated by 10% sodium dodecyl sulfate polyacrylamide gel electrophoresis (SDS-PAGE) and then transferred to polyvinylidene difluoride (PVDF) membranes, before immunoblotting with rabbit anti-Runx2 (D1L7F, CST, Danvers, MA, USA), rabbit anti-OPG (ab73400, Abcam, Cambridge, MA, USA), and mouse anti-RANKL (12A668, Abcam) primary antibodies. The rabbit anti-α/β-tubulin (2148, Abcam) was used as a loading control. Goat anti-rabbit (IRDye 800CW) and goat anti-mouse (IRDye 680CW) fluorochrome-conjugated secondary antibodies were purchased from Licor Biosciences (Lincoln, NB, USA). The protein bands were imaged on an Odyssey CLx near-infrared imager (Licor Biosciences) and quantified by Image Studio software (Licor Biosciences). Each band was normalized to its corresponding loading control band. The results were expressed as the percentage change compared to the untreated control.

### 2.9. Extracellular Matrix Mineralization Assay

The degree of extracellular matrix mineralization was determined by Alizarin Red S staining. Briefly, cells were seeded in 12-well plates at a density of 4 × 10^4^ cells per well in growth medium. After confluency, the culture medium was changed to differentiation medium only (control group) or differentiation medium containing TA2H and LPH (10, 100, and 1000 μg/mL, sample groups) for 15, 20, and 25 days. The medium was changed every 3 days. Then, the cultured wells were washed with PBS (pH 7.4) three times and fixed with 10% formalin for 1 h. After that, Alizarin Red S solution (40 mM, pH 4.2) was added to each well for 10 min at room temperature. The unbound stain was removed by rinsing with distilled water. The pictures were photographed under a light microscope. The quantitative analysis of mineralization degree was performed by eluting the bound stain with 10% (*w/v*) cetylpyridinium chloride solution in each well for 1 h, and the absorbance of solutions was measured by a microplate reader at a wavelength of 570 nm.

### 2.10. Detection of Signaling Pathway

The cells were seeded on 6 cm dishes at a concentration of 2 × 10^5^ cells per well and incubated in growth medium until confluency; then, the cells were serum-starved in α-MEM for 12 h. Then, 500 μg/mL of LPH was added to the culture medium before incubating for different time periods (0.5, 1, 2, 4, 8, 12, 24, and 36 h) to detect activation of the signaling pathway by Western blotting. Primary rabbit antibodies of extracellular signal regulated kinase 1/2 (ERK, 137F5, CST), phospho-ERK1/2 (Thr202/Try204, D13.14.4E, CST), p38 (AF1111, Beyotime), phospho-p38 (Thr180/Try182, AF5887, Beyotime), jun N-amino-terminal kinase (JNK, AJ518, Beyotime), phospho-JNK (Thr183/Try185, AF5860, Beyotime), Akt (C67E7, CST), phospho-Akt (Ser473, D9E, CST), and the loading control α/β-tubulin were used to examine the changes in MAPK and Akt signaling pathways.

### 2.11. Pathway Inhibitors Assay

The cells were seeded on six-well plates until confluency and were serum-starved in α-MEM for 24 h. Then, cells were pretreated with or without the p38 inhibitor SB203580 (5 μM, Beyotime), the ERK inhibitor FR180204 (2.5 μM, Beyotime), and the Akt inhibitor AZD5363 (2.5 μM, Beyotime) for 2 h, prior to coincubation with 500 μg/mL of LPH for 24 h. Cell lysates were evaluated for ALP activity and Runx2 expression.

### 2.12. Small Interfering RNA (siRNA) Knockdown of Runx2 Expression

The siRNA knockdown approach was as described by Zhang et al. [25] with slight modification. MC3T3-E1 cells were seeded on six-well plates with 1 × 10^5^ cells per well and incubated in growth medium for 24 h to reach ~50% confluency. Then, the cells were changed with Opti-MEM medium (Hyclone) and transfected with Runx2 siRNA (sc-37146, Santa Cruz, Dallas, TX, USA) or control siRNA using lentiviral particle (sc-37146-V, Santa Cruz) for 48 h. After siRNA transfection, cells were treated with 500 μg/mL of LPH for 24 h to collect whole-cell lysates, which were evaluated for ALP activity and Runx2 expression.

### 2.13. Statistical Analysis

All data are presented as the mean ± standard error of mean (SEM) of at least three independent experiments. Data were analyzed using one-way ANOVA with Dunnett’s post hoc test for comparison with control groups. SPSS 11.0 statistical software was used for the analyses. In all tests, statistical significance was set at *p* < 0.05.

## 3. Results

### 3.1. Amino-Acid Compositions of Whey Protein Hydrolysates

The amino-acid composition of TA2H and LPH is shown in Table A1. The Phe content dramatically reduced from 31.25 ± 0.67 to 1.17 ± 0.06 mg/g protein equivalent. The contents of glutamic acid, proline, tyrosine, and methionine were decreased after adsorption; meanwhile, the contents of branch-chain amino acids (valine, leucine, and isoleucine) in LPH were higher than in TA2H.

### 3.2. Whey Protein Hydrolysates Promote Cell Proliferation in MC3T3-E1 Osteoblasts

**Figure 1:**
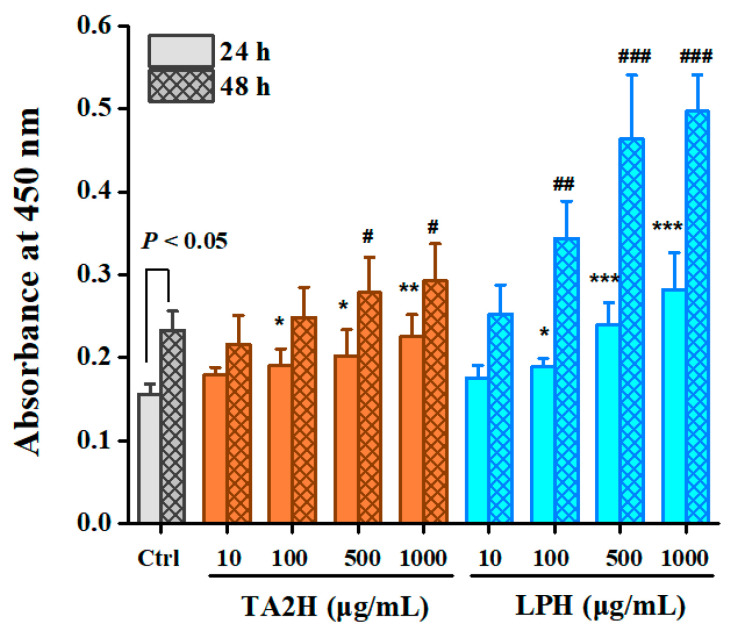
Effects of whey protein hydrolysates (TA2H and LPH) on cell proliferation in preosteoblast cells. MC3T3-E1 cells were serum-starved in 0.1% BSA/α-MEM for 12 h after seeding on 96-well plates for 24 h and were treated with serum starvation medium containing 0 (control group), 10, 100, 500, and 1000 μg/mL of TA2H and LPH for 24 or 48 h, prior to adding CCK-8. Data are represented as the mean ± SEM, *n* = 6. * (or #), ** (or ##), and *** (or ###) indicated *p* < 0.05, *p* < 0.01, and *p* < 0.001, as compared to the control group after 24 (or 48 h).

As shown in Figure 1, the two whey protein hydrolysates exerted a dose-related effect on osteoblast proliferation at 24 and 48 h. At the timepoint of 24 h, TA2H and LPH at concentrations of 100–1000 μg/mL showed significant increases in cell proliferation; moreover, LPH exerted significantly higher activities than TA2H at concentrations of 500–1000 μg/mL. At the timepoint of 48 h, the proliferation ratio further increased, in which 500–1000 μg/mL TA2H and 100–1000 μg/mL LPH exhibited significant promotion of proliferation relative to the control group of 48 h. The maximal stimulation was observed in the 1000 μg/mL LPH group at 48 h with over a twofold increase relative to the control. This group was followed by the 1000 μg/mL LPH group at 24 h, in which the proliferation was 1.7-fold greater than that in the control group. The concentration of 10 μg/mL was unable to boost the proliferation of MC3T3-E1 within 48 h. Overall, LPH possessed stronger stimulation activity in osteoblast proliferation than its original hydrolysate.

### 3.3. Whey Protein Hydrolysates Promote Cell Differentiation in MC3T3-E1 Osteoblasts

**Figure 2:**
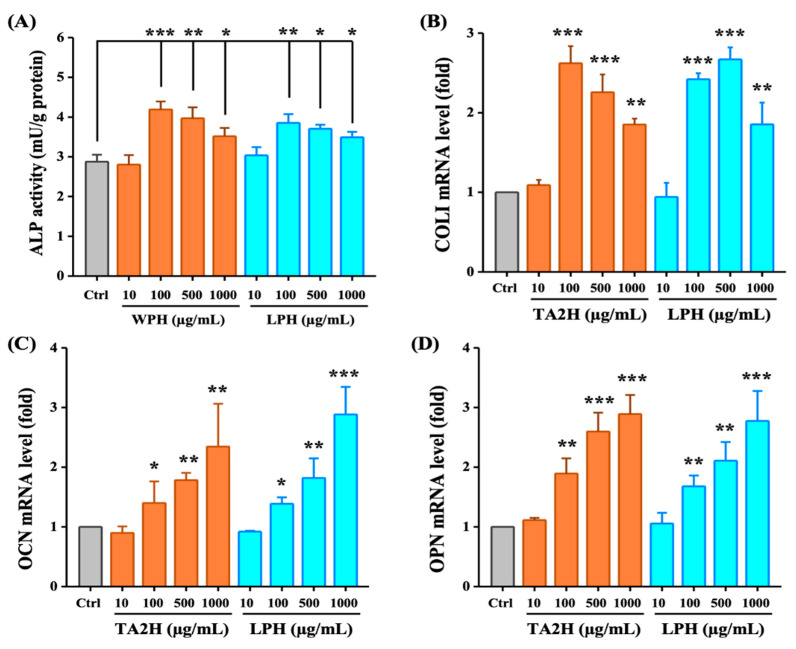
Effects of whey protein hydrolysates (TA2H and LPH) on cell differentiation in preosteoblast cells. MC3T3-E1 cells were seeded on six-well plates and were treated with growth medium containing 0 (control group), 10, 100, 500, and 1000 μg/mL of TA2H and LPH for 3 days to detect (**A**) ALP activity and (**B**) mRNA level of COLI, and for 6 days to detect the mRNA level of (**C**) OCN and (**D**) OPN. Data are represented as the mean ± SEM, *n* = 6. *, **, and *** indicated *p* < 0.05, *p* < 0.01, and *p* < 0.001, as compared to the control group.

**Figure A1:**
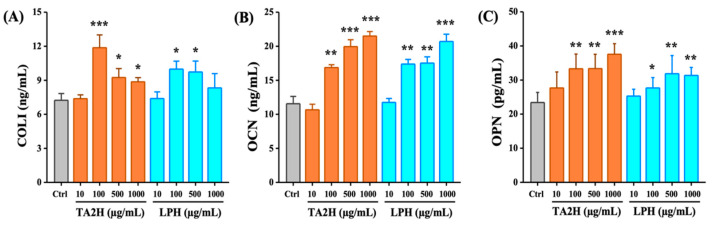
Effects of whey protein hydrolysates (TA2H and LPH) on osteoblastic differentiation markers in preosteoblast cells. MC3T3-E1 cells were seeded on six-well plates and were treated with 0 (control group), 10, 100, 500, and 1000 μg/mL of TA2H and LPH for 3 days to detect (**A**) COLI content in culture medium or cultured for 6 days to detect (**B**) OCN and (**C**) OPN content in culture medium. Data are the mean ± SEM from 4–6 independent experiments. *, **, and *** indicate *p* < 0.05, *p* < 0.01, and *p* < 0.001, as compared to the control group.

Type-I collagen (COLI) is the predominant organic matrix in bone, and ALP serves as a marker enzyme to synthesize the bone matrix. These two indices are widely used as phenotypic indicators of the early phase of osteoblast differentiation. OCN and OPN are strongly expressed in the late phase of osteoblast differentiation. As shown in Figure 2A, ALP activity significantly increased after treatment with 100–1000 μg/mL of TA2H and LPH, although the most effective stimulation dose was 100 μg/mL of TA2H. In addition, the upregulation of the mRNA expression of COLI was also dose-independent (Figure 2B). Whereas TA2H or LPH promoted the mRNA levels of OCN and OPN in a dose-dependent manner, and the concentration of 1000 μg/mL exerted the maximal promotion (Figure 2C,D). The trend of COLI, OCN, and OPN secretion was consistent with the mRNA levels (Figure A1A–C). A low concentration (10 μg/mL) of the two hydrolysates was insufficient to stimulate the differentiation of MC3T3-E1.

### 3.4. Whey Protein Hydrolysates Promote the Mineralization of Osteoblasts

**Figure 3:**
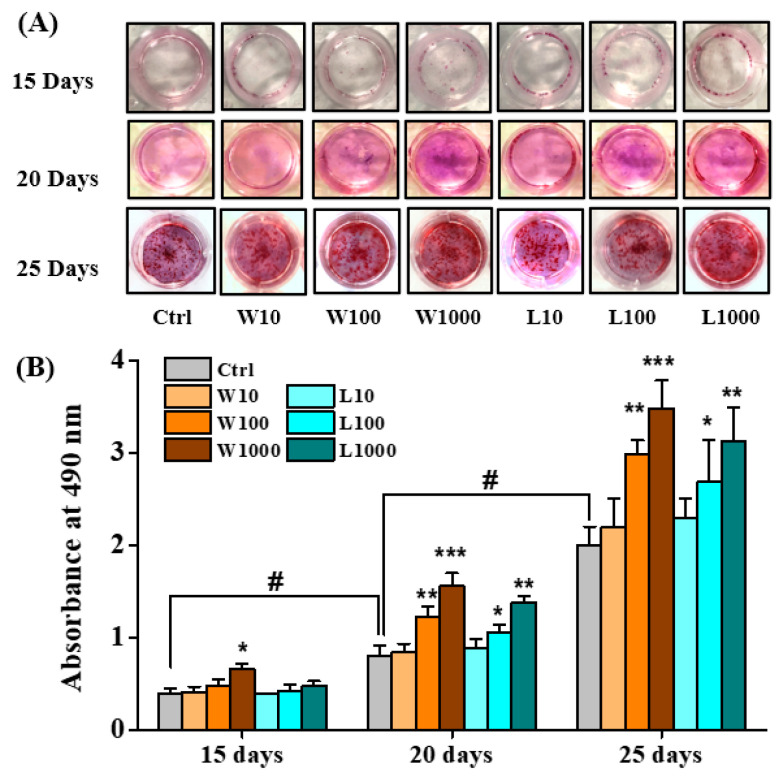
Effects of whey protein hydrolysates (TA2H and LPH) on mineralization in preosteoblast cells. MC3T3-E1 cells were incubated with differentiation medium containing 0 (control group), 10, 100, and 1000 μg/mL of TA2H and LPH for 15, 20, and 25 days prior to being stained with Alizarin Red S. (**A**) Representative images of mineralization were taken. (**B**) Absorbance at 490 nm was measured after distaining with cetylpyridinium chloride. Data are represented as the mean ± SEM, *n* = 3. *, **, and *** indicated *p* < 0.05, *p* < 0.01, and *p* < 0.001, as compared to the control group. # indicated *p* < 0.05 of the control group between day 15, 20 and 25.

During differentiation, osteoblasts secrete and calcify the extracellular matrix. Alizarin red S staining was used to evaluate the mineral deposition, and the dye was extracted and detected at 490 nm. As shown in Figure 3A,B, the calcium deposition and absorbance at 490 nm increased with incubation time in all groups. At day 15, only TA2H at a concentration of 1000 μg/mL had significantly higher absorbances than the control; at day 20 and 25, both TA2H and LPH at concentrations of 100–1000 μg/mL had significantly higher absorbances than the control. The 1000 μg/mL TA2H had maximal absorbance throughout the period; meanwhile, the 1000 μg/mL LPH showed a comparable effect on stimulating calcium deposition. However, the concentration of 10 μg/mL was insufficient to stimulate the mineralization of MC3T3-E1 during the whole incubation period. These results showed that TA2H and LPH could promote mineral deposition and calcium nodule formation in MC3T3-E1.

### 3.5. Whey Protein Hydrolysates Induce the Expression of Runx2 in Osteoblasts

**Figure 4:**
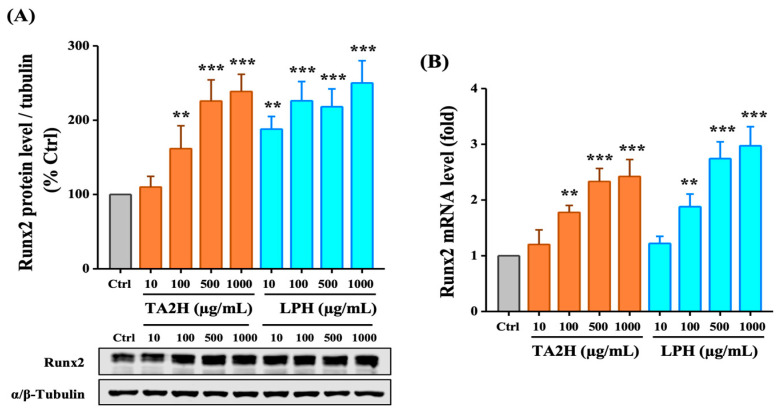
Effects of whey protein hydrolysates (TA2H and LPH) on Runx2 expression in preosteoblast cells. MC3T3-E1 cells were seeded on six-well plates and were treated with growth medium containing 0 (control group), 10, 100, 500, and 1000 μg/mL of TA2H and LPH for 72 h. (**A**) The relative protein expression of Runx2 was measured by Western blotting, and α/β-tubulin was used as the loading control. (**B**) The mRNA expression of Runx2 was determined by RT-PCR assay. Data are represented as the mean ± SEM, *n* = 3. **, and *** indicated *p* < 0.01 and *p* < 0.001, as compared to the control group.

Runx2 is the most extensively studied osteogenic transcription factor that plays an essential role in osteoblast differentiation and bone formation. As shown in Figure 4A, the level of Runx2 was increased 1.5- to 2.3-fold by 100–1000 μg/mL TA2H and 1.8- to 2.5-fold by 100–1000 μg/mL LPH after 72 h. The mRNA expression level of Runx2 was significantly increased after treatment with 100–1000 μg/mL TA2H and LPH, in which no difference was noted in the Runx2 levels of TA2H and LPH (Figure 4B). The results indicated that TA2H promoted the level of Runx2, which is a key factor for osteoblast differentiation.

### 3.6. Whey Protein Hydrolysates Promote the Ratio of OPG/RANKL in Osteoblasts

**Figure 5:**
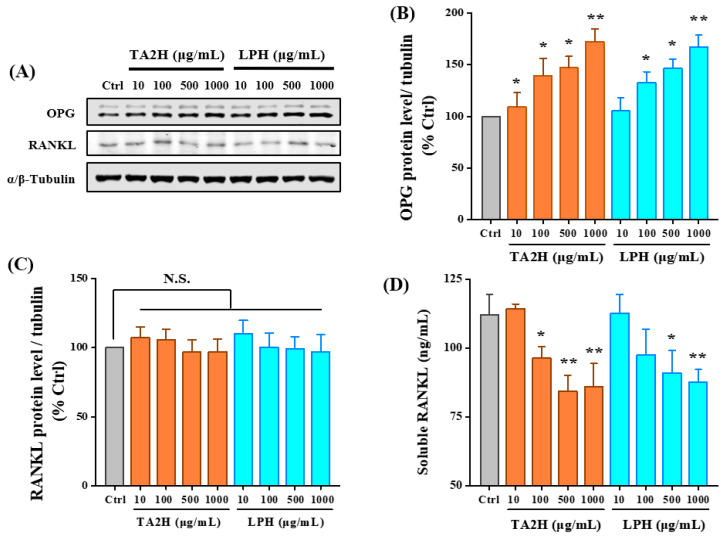
Effects of whey protein hydrolysates (TA2H and LPH) on OPG and RANKL expression in preosteoblast cells. MC3T3-E1 cells were seeded on six-well plates and were treated with growth medium containing 0 (control group), 10, 100, 500, and 1000 μg/mL of TA2H and LPH for 72 h prior to being lysed and immunoblotted for the Western blotting analysis of (**A**) OPG and (**B**) RANKL. The expression of α/β-tubulin was used as the loading control, and the fluorescence intensity of immunoblots was measured. All bands are shown in (**C**). The culture medium was evaluated for (**D**) soluble RANKL contents. Data are represented as the mean ± SEM, *n* = 3. * and ** indicated *p* < 0.05 and *p* < 0.01, as compared to the control group. N.S. indicated *p* > 0.05.

RANKL, secreted by osteoblasts, stimulates osteoclastogenesis by binding to RANK, while the soluble decoy receptor OPG competitively binds to RANKL and antagonizes its function. As shown in Figure 5B, the protein level of OPG was increased 1.7-fold by 1000 μg/mL TA2H and 1.6-fold by 1000 μg/mL LPH in a dose-dependent manner. However, only the soluble RANKL was significantly decreased after treatment with 100–1000 μg/mL TA2H and 500–1000 μg/mL LPH; the protein level of intracellular RANKL was insignificantly changed (Figure 5C,D). Our results indicated the potential of TA2H in preventing osteoclastogenesis through the reciprocal regulation of OPG production and RANKL secretion.

### 3.7. LPH Activates MAPK and AKT Pathways in Osteoblasts

**Figure 6:**
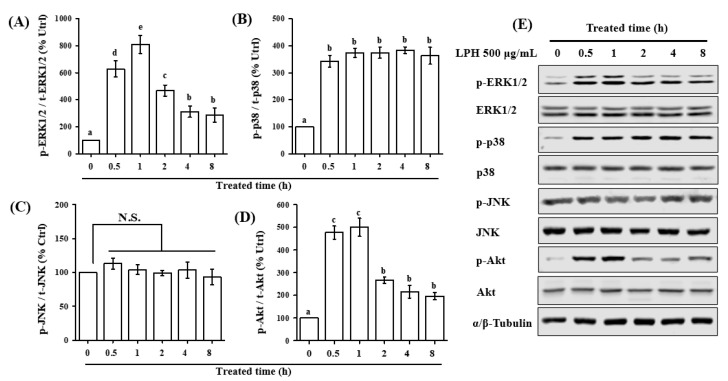
Effects of LPH on the activation of MAPK and Akt signaling in preosteoblast cells. MC3T3-E1 cells were seeded on 6 cm dishes until confluency and were serum starved in α-MEM for 12 h. Then, cells were treated with 500 μg/mL of LPH for different time periods. Cell lysates were immunoblotted for the Western blotting analysis of (**A**) p-ERK1/2/ERK1/2, (**B**) p-p38/p38, (**C**) p-JNK1/2/JNK1/2, and (**D**) p-Akt/Akt. The expression of α/β-tubulin was used as the loading control, and all bands are shown in (**E**). Fluorescence intensity of the immunoblots was measured. Data are represented as the mean ± SEM, *n* = 3. Means without the same lowercases indicated *p* < 0.05. N.S. indicated *p* > 0.05.

To reveal the underlying molecular mechanisms with regard to the osteogenic activity of LPH, we investigated whether LPH activated the three classic MAPKs (p38, ERK1/2, and JNK) and the major PI3K signaling target, Akt, in osteoblasts. The results in Figure 6 show that LPH activated the MAPK and AKT pathways in different manners. The treatment of 500 μg/mL LPH dramatically increased p38, ERK1/2, and Akt phosphorylation within the initial 0.5 h. The phosphorylation of ERK1/2 and Akt rapidly increased ~9- and ~5-fold within 1 h, respectively; thereafter, their phosphorylation levels quickly declined to the basal level (Figure 6A,D). For p38 phosphorylation (Figure 6B), LPH was shown to continuously stimulate p38 phosphorylation within 4 h (~5-fold); then, the phosphorylation of p38 declined to ~3.5-fold in 8 h. However, the phosphorylation of JNK was insignificantly affected by LPH (Figure 6C). These results indicated that LPH could activate Akt signaling, as well as the p38 and ERK1/2 pathways of MAPK signaling.

### 3.8. LPH Stimulates Osteoblast Differentiation through p38/Runx2 Pathway

**Figure 7:**
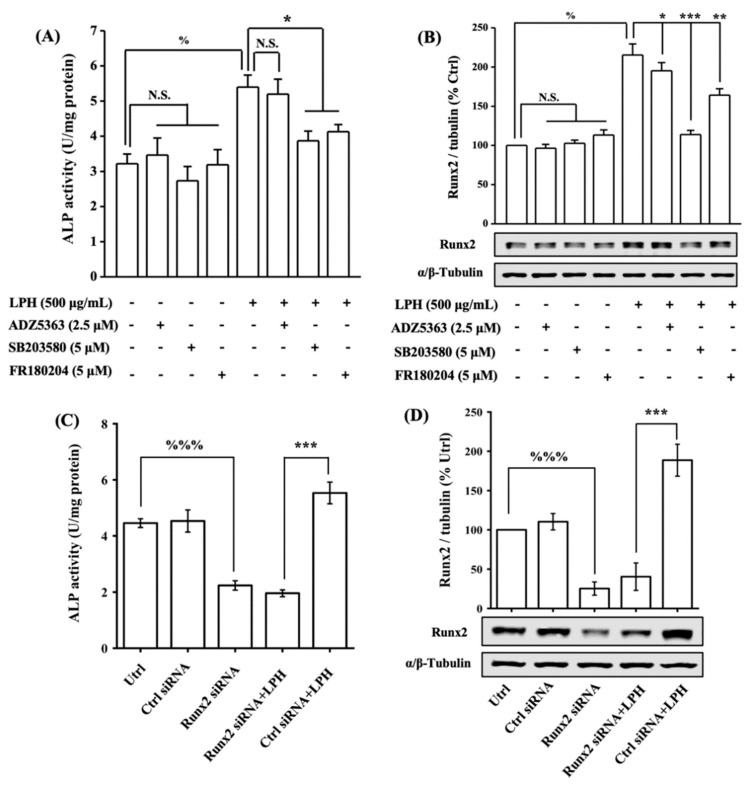
LPH stimulated osteoblast differentiation through p38/Runx2 pathway. (**A**,**B**) Effect of MAPK and Akt signaling specific inhibitors on LPH-induced ALP activity and Runx2 expression in MC3T3-E1 osteoblasts. Cells were seeded on six-well plates until confluency and were serum-starved in α-MEM for 12 h. Then cells were individually pretreated with or without 5 μM SB203580 (p38 MAPK inhibitor), 5 μM FR180204 (ERK1/2 MAPK inhibitor), and 2.5 μM ADZ5363 (Akt inhibitor) for 2 h, prior to coincubation with 500 μg/mL of LPH for 24 h to detect (**A**) ALP activity and (**B**) Runx2 expression. (**C**,**D**) Effect of Runx2 knockdown on LPH-induced ALP activity and Runx2 expression in MC3T3-E1 osteoblasts. Cells were seeded on six-well plates until 50% confluency and were transfected with Runx2 siRNA or control (Ctrl) siRNA for 48 h, prior to coincubation with 500 μg/mL of LPH for 24 h to detect (**C**) ALP activity and (**D**) Runx2 expression. The expression of α/β-tubulin was used as the loading control to Runx2. Data are represented as the mean ± SEM, *n* = 3. % and %%% indicate *p* < 0.05 and *p* < 0.001, as compared to the control group. *, ** and *** indicate *p* < 0.05, *p* < 0.01 and *p* < 0.001, as compared to the LPH treated group. N.S. indicated *p* > 0.05. N.S. indicated *p* > 0.05.

To further decipher the signal transduction pathway mediating the LPH-induced osteoblast differentiation, we applied inhibitors of the signaling pathways, namely, SB203580 as the p38 MAPK inhibitor, FR180204 as the ERK1/2 MAPK inhibitor, and ADZ5363 as the Akt inhibitor. As shown in Figure 7A,B, the treatment of 500 μg/mL LPH in MC3T3-E1 for 24 h significantly increased ALP activity and Runx2 expression by 62.53% and 117.46%, respectively; meanwhile, the pretreatment of individual pathway inhibitors for 2 h had no significant effect on ALP activity and Runx2 expression. The blocking of the p38 and ERK1/2 pathways significantly decreased the LPH-activated ALP activity by 84.33% and 78.69%, respectively, while the blockage of the Akt activity caused a minor reduction in ALP activity (14.50%). Moreover, the blocking of p38 signaling significantly reduced the activated Runx2 expression by 55.53%, thus causing it to drop to the basal line. Meanwhile, the ERK1/2 and Akt inhibitors inhibited 23.81% and 14.07% of the Runx2 expression, respectively. The siRNA knockdown approach was further used to examine the effect of Runx2 knockdown on LPH-induced osteogenesis. As shown in Figure 7C,D, MC3T3 E1 cells transfected with Runx2 siRNA or control siRNA, and Runx2 siRNA transfection effectively decreased the basal level of Runx2 expression and ALP activity. The LPH-induced Runx2 expression and ALP activity were significantly inhibited by Runx2 knockdown. These combined results indicated the role of Runx2 in mediating LPH-induced osteogenesis.

## 4. Discussion

The development of the osteoporotic state involves multiple mechanisms in which the disordered homeostasis between bone formation and bone resorption initiates the breakdown of the bone matrix [14]. Although systematic reviews and meta-analyses illustrated that the reduced BMD of PKU individuals was within the normal range according to the standard definitions of low BMD [9,10,11], numerous individual studies have demonstrated the PKU individuals exerted lower BMD, and certain etiologies remain unclear [9,10,11]. The prepared LPH in our study contained minimum Phe, which was consistent with our previous study [26]. The contents of glutamic acid, proline, tyrosine, and methionine in LPH were lower than those of TA2H and original whey protein. In particular, the content of tyrosine, as one of the indispensable amino acids and the converting product of Phe, would need to be supplemented in a future formulation. As whey protein possesses many bioactive components that benefit the musculoskeletal system, it is beneficial to investigate the bone anabolic potency of the prepared whey protein hydrolysates. Osteoblasts not only act as the most anabolic bone cell synthesizing matrix proteins, but also regulate the formation of osteoclasts by secreting M-CSF, RANKL, and OPG [15]. Our preliminary experiments showed that the proliferative activity of 100 μg/mL TA2H in MC3T3-E1 preosteoblasts was comparable to that of 10 μg/mL bovine LF (data not shown). Thus, we further investigated the osteogenic activities of TA2H and LPH in MC3T3-E1 cells. As shown in the present study, MC3T3-E1 preosteoblasts treated with 100–1000 μg/mL TA2H and LPH significantly promoted cell proliferation, differentiation, and mineralization; moreover, whey protein hydrolysates inhibited bone resorption by promoting the OPG/RANKL ratio. The effects of LPH on the proliferation and differentiation of osteoblasts were comparable to those of the original TA2H, and the p38-dependent Runx2 pathway possibly contributed to the osteogenic activity.

The recruitment of preosteoblasts at the bone surface initiates bone formation; moreover, the proliferation of osteoblasts is the prerequisite to reverse the predominant activity from osteoclasts into osteoblasts [15]. The TA2H and LPH, ranging from 100 μg/mL to 1000 μg/mL, promoted osteoblast proliferation in a dose-dependent manner. Jang [17] reported that MC3T3-E1 cells treated with 500 μg/mL whey protein hydrolysate for 24 h had a 1.3-fold increase in terms of cell viability. Two ACE-inhibitory peptides derived from whey protein (YLLF and YVEEL) significantly promoted osteoblast viability at 50 ng/mL [20]. Similarly, our results showed that the prepared whey protein hydrolysates stimulated osteoblast proliferation. In addition, bovine LF was found to shorten the cell cycle by increasing the length of the G2/M and S phases of MC3T3-E1 cells [30], while the pea-derived bioactive peptide LRW was observed to increase the migration rate of osteoblasts [24]. These results indicate that other approaches can be further examined to investigate the activity of whey protein hydrolysates in preosteoblasts.

After the recruitment, preosteoblasts undergo the differentiation process to become osteoblasts and produce extracellular bone matrix [15]. The TA2H and LPH dose-dependently activated OCN and OPN contents, and the maximal stimulation of ALP activity and COLI content was observed at 100 μg/mL of TA2H and LPH. The inconsistency might be due to the differences in the sensibility and accumulation of osteoblast-specific markers. Matrix mineralization is the last and longest stage of bone formation with the accumulation of the extracellular matrix. After cultivation for 20–25 days, TA2H and LPH significantly increased the formation of calcium nodules in MC3T3-E1. The dose- and time-dependent tendency toward mineralization was consistent with the late-phase differentiation markers (OCN and OPN), thus indicating the potential benefits of TA2H in bone matrix accumulation.

Runx2 has been identified as a master regulator of skeletogenesis [23]. Homozygous Runx2 knockout or Runx2 carboxy-terminus-truncated mice displayed severe skeletal defects and hypertrophic cartilage [31,32]. Two dominant osteoblastic signaling pathways, which transform the growth factor-beta (TGF-β)/bone morphogenic protein (BMP) pathway and Wnt/β-catenin pathway, converge at the transcription of the *Runx2* gene and coordinate with Runx2 to control the transcription of osteoblastic genes, such as *col*, *alp*, *bsp*, *ocn*, and *opn* [33]. Additionally, the regulatory role of Runx2 in osteoblasts extends throughout the proliferation and differentiation periods. In our study, TA2H and LPH dose-dependently augmented Runx2 expression. The knockdown of Runx2 using Runx2 siRNA almost completely blocked the LPH-induced differentiation, suggesting that Runx2 acts as a key target of whey protein hydrolysate-induced osteogenesis. However, whether the phosphorylation of Runx2 was mediated by whey protein hydrolysates was unclear. Runx2 has multiple phosphorylation sites, and its phosphorylation events at different residues led to different conformational changes, thereby conferring inhibitory or stimulatory effects [34]. For example, fibroblast growth factor 2 (FGF2) induces the phosphorylation of Runx2 at the C-terminal region through ERK1/2 MAPK-stimulated Runx2 activity [35], while the phosphorylation of Runx2 at Ser104 and Ser451 inhibits its activity [36]. Therefore, the effects of whey protein hydrolysates on the phosphorylation level of Runx2 should be explored in future studies.

The investigation into the molecular mechanisms with regard to the osteogenic activity of LF supported the potential of LPH to be used as a new osteoinductive formula reagent in the future. Akt and MAPK signaling pathways are deeply involved in skeletal development and bone homeostasis [21,22]. Runx2 reportedly intertwines with the PI3K/Akt axis, and they reciprocally activate one another, thereby promoting osteogenesis [37]. Among the three classic MAPKs, ERK and p38 signaling can boost the transcription and phosphorylation of Runx2 [38]. In particular, the activation of p38 not only directly promotes the phosphorylation of osterix at the residues of Ser77 and Ser33 [39], but also indirectly increases the transcriptional ability of *osterix* by recruiting histone acetyltransferase p300 [40]. Furthermore, the interaction of osterix and Runx2 can increase their transcriptional power [41]. In our study, LPH activated the phosphorylation of p38, ERK1/2, and Akt, with the stimulation on p38 phosphorylation not being the maximal but being the longest-lasting. SB203580 and FR180204 significantly inhibited the promoted ALP activity by LPH, and SB203580 almost completely blocked the LPH-induced Runx2 expression. Meanwhile, the treatment of FR180204 and ADZ5363 reserved part of the promotion. Suzuki [42] reported that the blocking of the p38 pathway with 10 μM SB203580 in MC3T3-E1 cells inhibited ALP activity, but only higher doses (10–30 μM) of SB203580 inhibited COL and OCN secretion; this result suggested that the p38 pathway was deeply involved in osteoblastic differentiation. The activation of ERK1/2 MAPK signaling in osteoblasts contributed to the mitogenic effect of LF [28], while the activation of p38 MAPK pathways, and the downstream phosphorylation of Runx2 contributed to LF-induced osteoblast differentiation [25]. PI3K/Akt signaling in osteoblasts was also activated by LF, whereas PI3K and ERK1/2 MAPK signaling did not contribute to LF-induced osteoblast survival. A casein-derived peptide NAVPITPTL was found to induce the osteoblastic gene expression through Akt but not through the ERK signaling cascade [43]. Our results indicated that LPH stimulated osteoblast differentiation mainly through p38-dependent Runx2 activation and that Akt signaling might play a minor role in LPH-activated osteoblast differentiation.

Increased circulating RANKL accompanied by concurrently enhanced osteoclastogenesis was observed in the peripheral blood mononuclear cells isolated from PKU individuals [12,13]. RANKL exists in intracellular and extracellular (or soluble) form, with extracellular RANKL produced from the shedding of membrane-bound RANKL by matrix metalloproteinase 14 (MMP14) or disintegrin and metalloproteinase 10 (ADAM10) [44]. Free soluble RANKL is not bound to OPG and easily combines with its receptor RANK [45]. Therefore, circulating/soluble RANKL is a crucial mediator of osteoclastogenesis. In our study, LPH significantly inhibited the soluble RANKL and increased the intracellular OPG expression, thus suggesting its inhibitory effect on bone resorption. As the expression of intracellular RANKL was insignificantly affected, we hypothesize that whey protein hydrolysates inhibited RANKL-induced osteoclastogenesis mainly through binding to extracellular RANKL.

Our results showed that, under the same culture conditions, LPH had comparable osteogenic activity to TA2H, thus indicating that LPH as a whole had the capacity to promote osteoblast differentiation. In conclusion, for the first time, we reported the osteoinductive potential of LPH and suggested its application as a functional protein substitute benefiting the bone health of PKU individuals. The activated p38/Runx2 signaling contributed to the LPH-stimulated osteoblast differentiation, while the involvement of other osteoblastic pathways has yet to be deciphered. Further study is also warranted to verify the in vivo efficacy of LPH.

## Figures and Tables

**Table 1:** Primers sequences used for RT-PCR.

Gene Name	Primer Sequence (5′ to 3′)
*Runx2*	Forward: CCTTCAAGGTTGTAGCCCTC
	Reverse: GGAGTAGTTCTCATCATTCC
*COLI*	Forward: CAAGATGTGCCACTCTGACT
	Reverse: TCTGACCTGTCTCCATGTTG
*OCN*	Forward: AGACTCCGGCGCTACCTTGG
	Reverse: CGGTCTTCAAGCCATACTGG
*OPN*	Forward: TCAGGCATGTCCCTCGGTAT
	Reverse: TGGCAGGTAGGTATGGTAGT
*β-Actin*	Forward: TTGCTGACAGGATGCAGAAG
	Reverse: ACATCTGCTGGAAGGTGGAC

## Abbreviations

ALP	Alkaline phosphatase
BMD	Bone mineral density
BSP	Bone sialoprotein
COLI	Type-I collagen
ELISA	Enzyme-linked immunosorbent assay
ERK	Extracellular signal regulated kinase 1/2
FBS	fetal bovine serum
JNK	Jun N-amino-terminal kinase
LF	Lactoferrin
MAPK	Mitogen-activated protein kinase
α-MEM	α-Minimum essential medium
OCN	Osteocalcin
OPG	Osteoprotegerin
OPN	Osteopontin
Phe	Phenylalanine
PI3K/Akt	Phosphatidylinositol 3-kinase/protein kinase B
PKU	Phenylketonuria
RANK	Receptor activator of nuclear factor-κB
RANKL	Receptor activator of nuclear factor-κB ligand
Runx2	Runt-related transcription factor 2
WPC	Whey protein concentrate

## Appendix A

**Table A1:** Amino-acid compositions of whey protein hydrolysates.

Amino-Acid Composition (g/100 g)	TA2H	LPH
Alanine	4.32 ± 0.06	4.41 ± 0.07
Aspartic acid	7.70 ± 0.24	5.96 ± 0.12
Glycine	1.13 ± 0.06	0.81 ± 0.04
Glutamic acid	11.89 ± 0.51	6.79 ± 0.24
Serine	2.75 ± 0.04	2.41 ± 0.02
Histidine	2.07 ± 0.13	2.15 ± 0.10
Arginine	2.78 ± 0.11	2.50 ± 0.13
Threonine	4.86± 0.05	4.42 ± 0.20
Proline	5.16 ± 0.08	1.96 ± 0.11
Tyrosine	2.43 ± 0.03	0.78 ± 0.02
valine	3.86 ± 0.11	5.98 ± 0.23
Methionine	2.36 ± 0.05	1.87 ± 0.24
Cystine	0.44 ± 0.03	0.35 ± 0.02
Isoleucine	4.79 ± 0.22	5.80 ± 0.15
Leucine	7.62 ± 0.44	9.05 ± 0.37
Phe	2.33 ± 0.11	0.07 ± 0.02
Protein content ^1^ (g/100 g)	74.48 ± 1.05	63.34 ± 1.67
Phe content ^2^ (mg/g protein equivalent)	31.25 ± 0.67	1.17 ± 0.06

^1^ The protein content was measured using the Kjeldahl method. ^2^ The Phe content was calculated as the Phe concentration in relation to the protein content.
